# Supplementary material for: Matrix Metalloproteinase 9 Induced in Esophageal Squamous Cell Carcinoma Cells via Close Contact with Tumor-Associated Macrophages Contributes to Cancer Progression and Poor Prognosis
Source: Cancers (Basel). 2023 May 30;15(11):2987. doi: 10.3390/cancers15112987 (PMC10252039; doi:10.3390/cancers15112987)
Supplement: Supplementary file 1 [file cancers-15-02987-s001.zip › cancers-2369570-supplementary.pdf]

Supplementary Materials

Matrix Metalloproteinase 9 Induced in Esophageal Squamous Cell Carcinoma Cells via Close Contact with Tumor-Associated Macrophages Contributes to Cancer Progression and Poor Prognosis

Shuichi Tsukamoto, Yu-ichiro Koma, Yu Kitamura, Kohei Tanigawa, Yuki Azumi, Shoji Miyako, Satoshi Urakami, Masayoshi Hosono, Takayuki Kodama, Mari Nishio, Manabu Shigeoka, Hiroshi Yokozaki

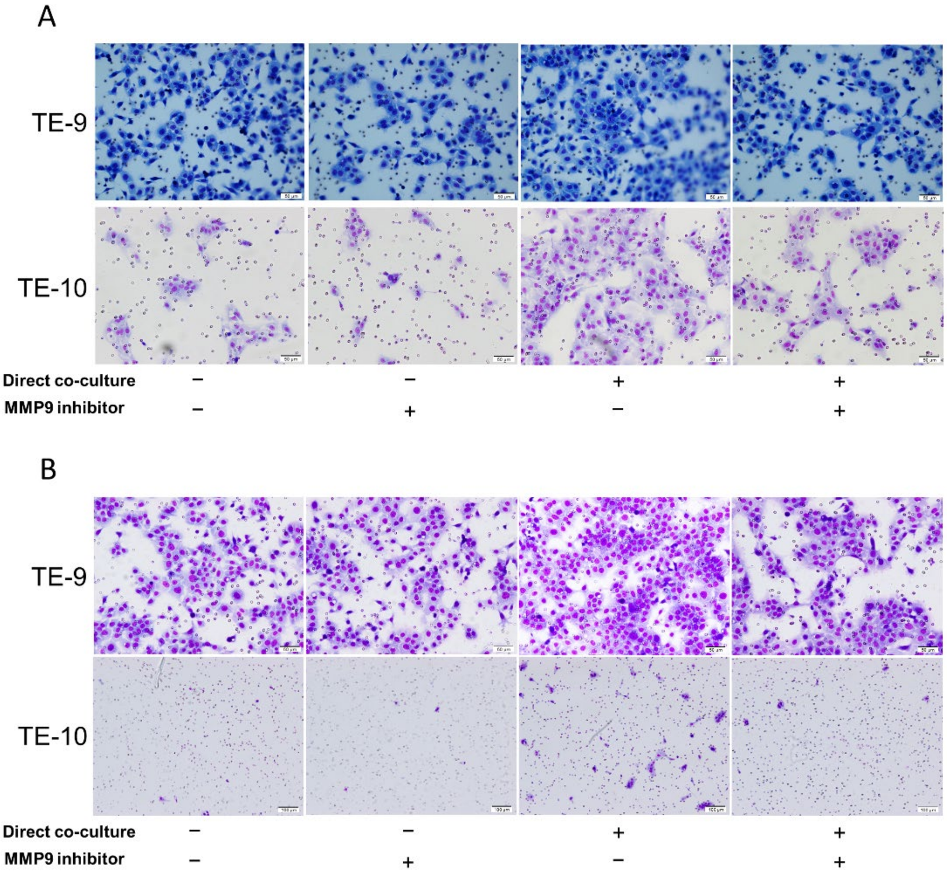

Figure S1. Representative images of transwell migration (A) and invasion (B) assays using TE-9 and TE-10 shown in Figure 4.

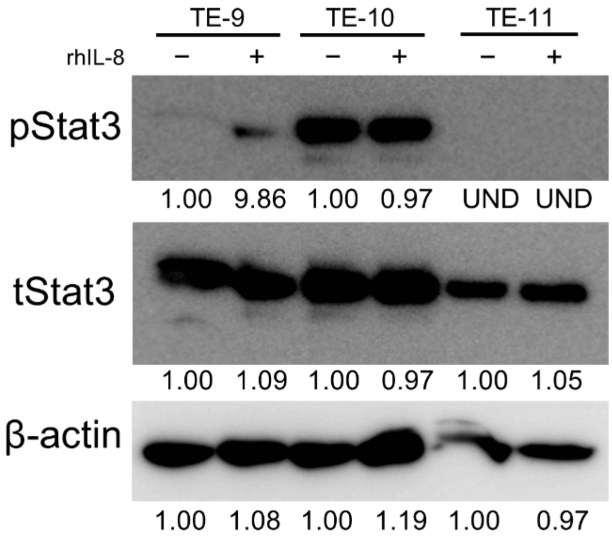

Figure S2. rhIL-8 treatment does not induce Stat3 phosphorylation in all ESCC cell lines used. rhIL-8 treatment (100 ng/mL, for 24 h) promoted Stat3 phosphorylation only in TE-9 cells among the three ESCC cell lines used. Densities of bands were calculated using the ImageJ software and expressed as relative values. Density of untreated control cells in each cell line was set as 1.00. p: phosphorylated; t: total; UND: undetected.

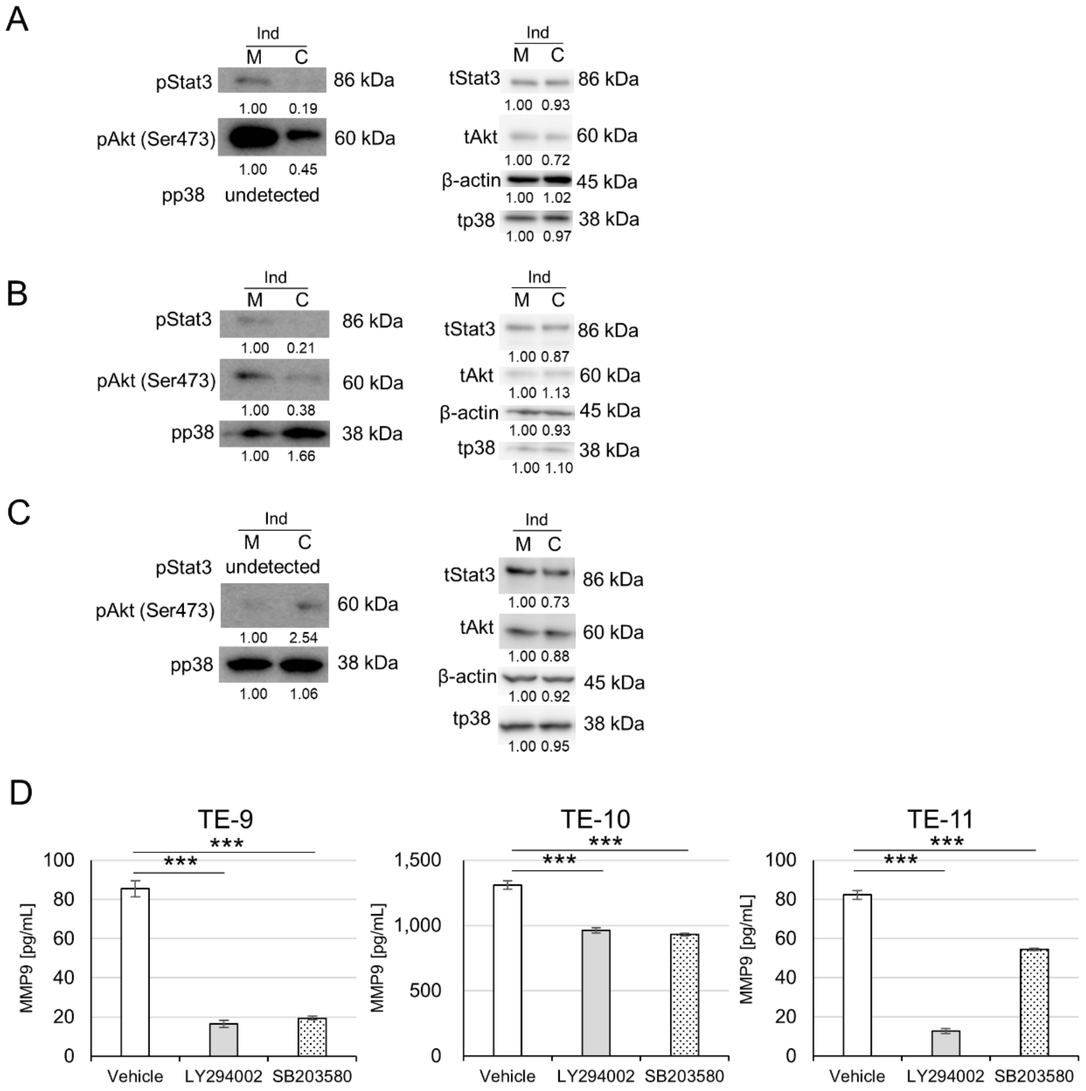

Figure S3. Akt and p38 MAPK pathways also involve in MMP9 secretion in basal state ESCC cells. A-C: Akt and p38 phosphorylations after indirect co-culture with macrophages differed in cell lines examined. The band density of monocultured conditions in each cell line was set as 1.00. A, TE-9; B, TE-10; C: TE-11; Ind: indirect; M: monoculture; C: co-culture; p: phosphorylated; t: total. D: LY294002 (PI3K inhibitor; 10 μM) or SB203580 (p38 inhibitor; 10 μM) treatment for 24 h decreased MMP9 secretion from ESCC cells. ***$p < 0.05$.

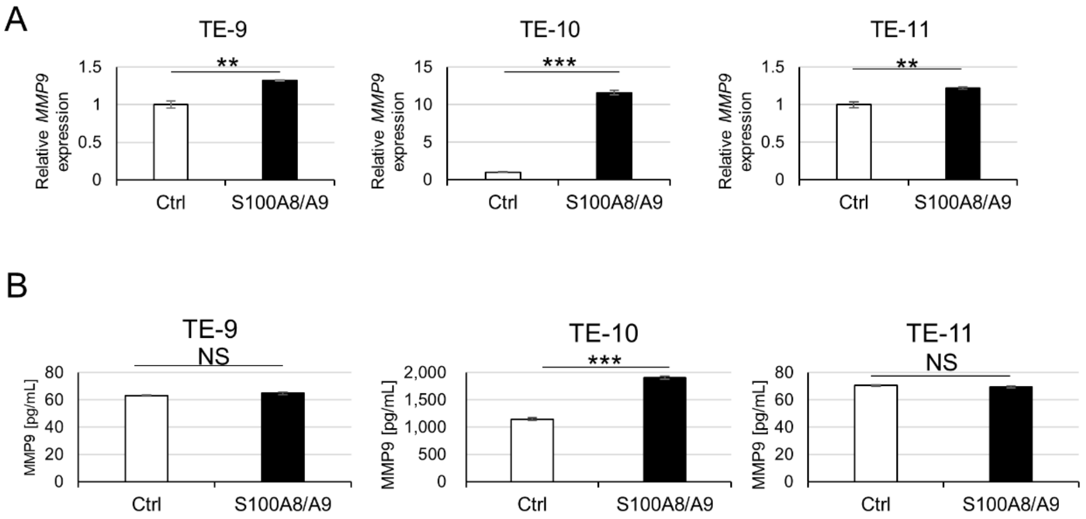

Figure S4. S100A8/A9 as a partial inducer of MMP9 in ESCC cells. A, B: Treatment with rhS100A8/A9 (10 µg/mL, for 24 h) upregulated *MMP9* mRNA expression in all three ESCC cell lines (A) but only increased MMP9 secretion from TE-10 (B). Data are expressed as mean ± SEM; $^{**}p < 0.01$, $^{***}p < 0.001$. NS, not significant.

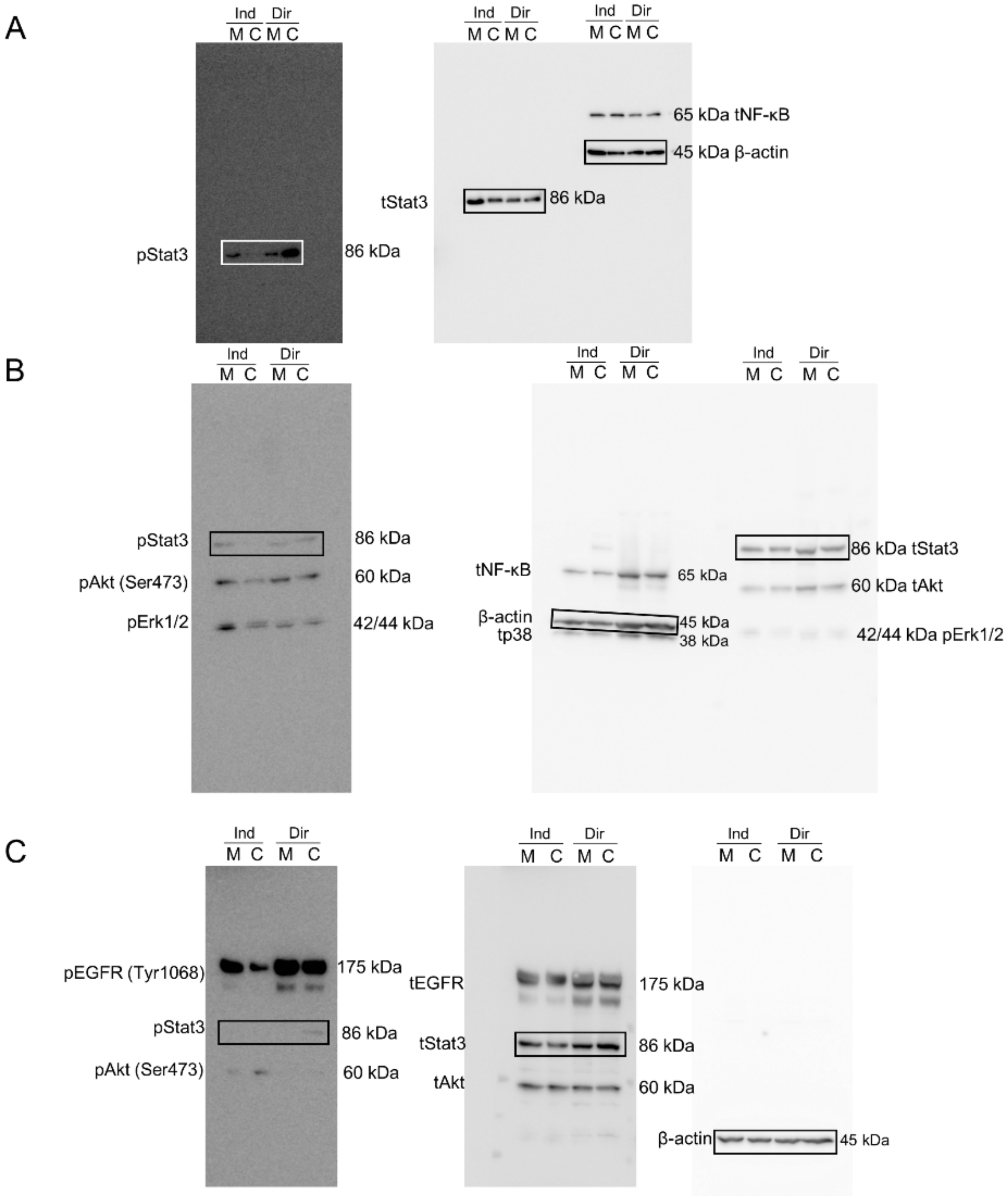
A
Ind Dir
M C M C
pStat3
86 kDa
tStat3
86 kDa
65 kDa tNF-κB
45 kDa β-actin
B
Ind Dir
M C M C
pStat3
86 kDa
pAkt (Ser473)
60 kDa
pErk1/2
42/44 kDa
tNF-κB
65 kDa
β-actin
45 kDa
tp38
38 kDa
86 kDa tStat3
60 kDa tAkt
42/44 kDa pErk1/2
C
Ind Dir
M C M C
pEGFR (Tyr1068)
175 kDa
pStat3
86 kDa
pAkt (Ser473)
60 kDa
tEGFR
175 kDa
tStat3
86 kDa
tAkt
60 kDa
β-actin
45 kDa

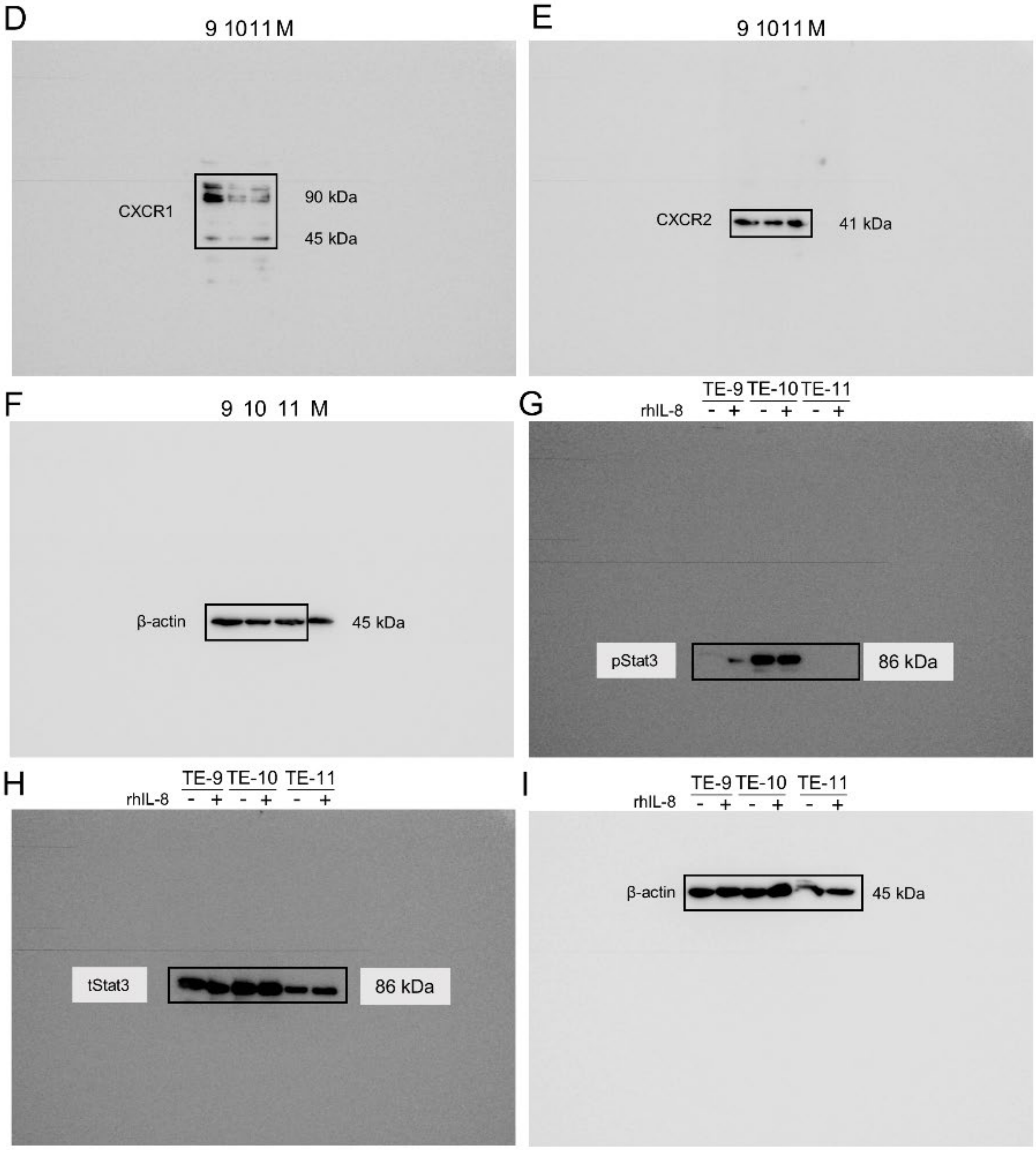
D
9 1011 M
CXCR1
90 kDa
45 kDa
E
9 1011 M
CXCR2
41 kDa
F
9 10 11 M
β-actin
45 kDa
G
TE-9 TE-10 TE-11
rhIL-8 - + - + - +
pStat3
86 kDa
H
TE-9 TE-10 TE-11
rhIL-8 - + - + - +
tStat3
86 kDa
I
TE-9 TE-10 TE-11
rhIL-8 - + - + - +
β-actin
45 kDa

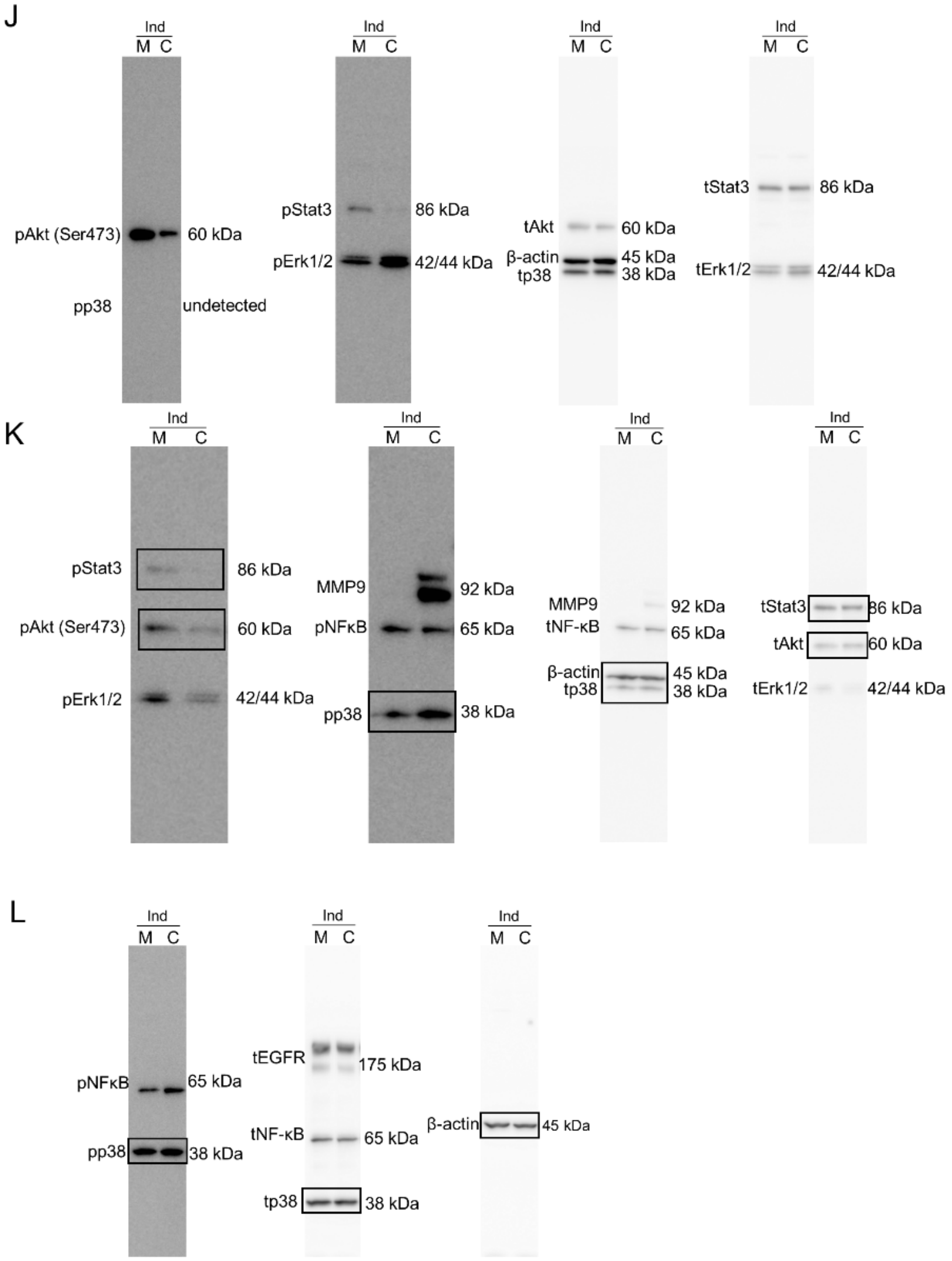

Figure S5. Whole images of western blot shown in Figure 3A, S3 (A-C), Figure 6B (D-F), Figure S2 (G-I), and Figure S3 (J-L). A-C: The membranes were reused several times to identify other bands. The pre-stained markers used were of three colors that did not contain chemiluminescent substances; thus, the markers are not visible in the presented raw data. Rectangles indicate the bands shown in main figures. A: TE-9; B: TE-10; C: TE-11. Ind: indirect; Dir: direct; M: monoculture; C: co-culture; p: phosphorylated; t: total. D-F: 9, TE-9; 10, TE-10; 11, TE-11; M, PBMo-derived macrophage. J-L: Ind, indirect; M, monoculture; C, co-culture.

Table S1. Fold changes of spot densities in cytokine array analysis, listed in descending order.

Analytes	Fold changes (Co/Mono)	Analytes	Fold changes (Co/Mono)	Analytes	Fold changes (Co/Mono)	Analytes	Fold changes (Co/Mono)
MMP9	1.94	CCL7	1.05	IL-17a	0.93	HGF	0.79
IL-8	1.61	CD40 ligand	1.05	CXCL10	0.92	Dkk-1	0.79
PDGF-AA	1.58	Cystatin C	1.04	RAGE	0.91	M-CSF	0.77
Angiogenin	1.42	CXCL4	1.04	CCL19	0.91	TGF-α	0.77
uPAR	1.38	IL-5	1.04	FGF19	0.91	IL-19	0.76
CCL3/CCL4	1.27	Fas ligand	1.03	VEGF	0.90	C-reactive protein	0.75
IL-2	1.26	MIF	1.03	IL-1R4	0.90	TIM3	0.75
Cripto-1	1.24	IL-3	1.03	IL-22	0.88	Thrombospondin-1	0.72
IGFBP2	1.23	GM-CSF	1.02	TfR1	0.88	Adiponectin	0.71
Vitamin D BP	1.21	CXCL11	1.02	BDNF	0.88	Leptin	0.71
Chitinase 3-like 1	1.20	IL-32	0.99	Angiopoietin-1	0.87	IL-31	0.71
CCL17	1.19	IL-6	0.98	PAI-1	0.86	IL-1β	0.70
CCL2	1.18	IL-27	0.98	IL-4	0.86	Endoglin	0.69
IL-12 p70	1.18	FGF7	0.97	IL-34	0.85	CD30	0.68
IL-23	1.17	ENA-78	0.97	Relaxin2	0.84	IL-16	0.66
BAFF	1.14	CCL20	0.97	LIF	0.84	IL-11	0.66
IL-24	1.14	IL-18Bpa	0.96	IL-33	0.84	EGF	0.64
Pentraxin 3	1.14	SHBP	0.96	C5a	0.83	Flt-3 ligand	0.64
CD14	1.13	Resistin	0.96	VCAM1	0.83	IL-13	0.63
OPN	1.12	Angiopoietin-2	0.95	PDGF-AB	0.83	IGFBP3	0.62
ICAM1	1.12	IFNγ	0.95	IL-15	0.82	GDF-15	0.60
TFF3	1.10	DPP-IV	0.95	IL-1α	0.81	FGF2	0.59
Kallikrein 3	1.09	CXCL12	0.94	IL-1ra	0.81	GROα	0.54
Growth hormone	1.09	IL-10	0.94	CD31	0.81	ApolipoproteinA-1	0.53
Complement factor D	1.08	MPO	0.94	EMMPRIN	0.80	G-CSF	0.49
CXCL9	1.07	CCL5	0.93	RBP4	0.80	TNF-α	0.45
NGAL	1.06						

Table S2. Relative gene expression changes of the ten analytes shown in Figure 1A, as demonstrated by cDNA microarray.

Accession number	Gene description	Symbol	Log2 ratio (Co/Mono)
NM_004994.2	matrix metallopeptidase 9	*MMP9*	4.12
NM_000583.3	group-specific component (vitamin D binding protein)	*GC*	0.45
NM_001005377.2	plasminogen activator, urokinase receptor	*PLAUR*	0.26
NG_029727.1	platelet-derived growth factor alpha polypeptide	*PDGFA*	0.07
NM_000586.3	interleukin 2	*IL2*	0.00
NM_001313992.1	insulin like growth factor biding protein 2	*IGFBP2*	-0.18
NM_002983.2	C-C motif chemokine ligand 3	*CCL3*	-0.19
NM_001097577.2	angiogenin, ribonuclease, RNase A family, 5	*ANG*	-0.26
NM_001079530.1	cripto, FRL-1, cryptic family 1B	*CFC1B*	-0.81
NM_000584.3	C-X-C motif chemokine ligand 8	*CXCL8*	-0.89

Table S3. Top 20 upregulated pathways in TE-11 cells after direct co-culture with macrophages.

Upregulated pathways	p-value (Comparison)	Matched Entities (Comparison)	Pathway Entities of Experiment Type (Comparison)
Hs_TYROBP_Causal_Network_WP3945_90843	2.25E-07	16	60
Hs_Neutrophil_degranulation_WP4049_93311	1.09E-06	54	480
Hs_GPCR_downstream_signaling_WP1824_93957	1.79E-06	86	958
Hs_Vitamin_D_Receptor_Pathway_WP2877_94793	5.92E-06	27	186
Hs_Mammary_gland_development_pathway_-_Involution_(Stage_4_of_4)_WP2815_88702	7.60E-06	6	10
Hs_GPCRs,_Class_A_Rhodopsin-like_WP455_96878	8.78E-06	34	262
Hs_Assembly_of_collagen_fibrils_and_other_multimeric_structures_WP2798_93410	5.06E-05	11	46
Hs_Allograft_Rejection_WP2328_97563	1.01E-04	16	99
Hs_Interleukin-4_and_13_signaling_WP4066_93379	1.68E-04	26	210
Hs_Human_Complement_System_WP2806_97328	2.74E-04	16	136
Hs_Matrix_Metalloproteinases_WP129_72054	3.61E-04	8	31
Hs_Microglia_Pathogen_Phagocytosis_Pathway_WP3937_94208	4.74E-04	9	40
Hs_Immunoregulatory_interactions_between_a_Lymphoid_and_a_non-Lymphoid_cell_WP1829_96981	5.97E-04	16	278
Hs_MTHFR_deficiency_WP4288_97729	9.53E-04	5	18
Hs_Antimicrobial_peptides_WP4101_93538	9.90E-04	9	48
Hs_Degradation_of_the_extracellular_matrix_WP2774_93614	0.001206	12	76
Hs_Macrophage_markers_WP4146_94392	0.001246	4	9
Hs_Toll-Like_Receptors_Cascades_WP2775_93988	0.001293	7	32
Hs_Regulation_of_TLR_by_endogenous_ligand_WP4069_93389	0.00136	5	16
Hs_Complement_cascade_WP1798_93740	0.001378	10	145

Pathway analysis was perfomed by comparing gene expression changes identified with microarray analysis and deposited molecular pathways (WikiPathways). Top 20 pathways included “Matrix_Metalloproteinases” and “Degradation_of_the_extracellular_matrix”, which motivated us to investigate MMP9 in the present study.

Table S4. Correlations between expressions of IL-8 and its receptors (CXCR1 and CXCR2) in cancer nests and MMP9 at the invasive front, as analyzed by immunohistochemistry.

	Number of cases	Cancer nest expression of MMP9		*p*-value
		Negative (n = 50)	Positive (n = 19)	
IL-8 expression[a]				
Negative	14	13	1	0.056
Positive	55	37	18	
CXCR1 expression[a]				
Low	40	28	12	0.591
High	29	22	7	
CXCR2 expression[a]				
Low	39	33	6	0.010*
High	30	17	13	

[a] IL-8, CXCR1, and CXCR2 expressions were evaluated in cancer nests and classified as low and high compared to expressions of the corresponding nonneoplastic esophageal epithelia [8]. Data were analyzed with a Chi-square test. $*p < 0.05$.
